# Comparison of the T2-star Values of Placentas Obtained from Pre-eclamptic Patients with Those of a Control Group: an Ex-vivo Magnetic Resonance Imaging Study

**DOI:** 10.4274/balkanmedj.2016.1472

**Published:** 2017-09-29

**Authors:** Nursel Yurttutan, Murat Bakacak, Betül Kızıldağ

**Affiliations:** 1 Department of Radiology, Kahramanmaraş Sütçü İmam University School of Medicine, Kahramanmaraş, Turkey; 2 Department of Obstetrics and Gynecology, Kahramanmaraş Sütçü İmam University School of Medicine, Kahramanmaraş, Turkey

**Keywords:** Ex vivo, magnetic resonance imaging, Placenta, pre-eclampsia, T2 star

## Abstract

**Background:**

Endotel dysfunction, vasoconstriction, and oxidative stress are described in the pathophysiology of pre-eclampsia, but its aetiology has not been revealed clearly.

**Aims:**

To examine whether there is a difference between the placentas of pre-eclamptic pregnant women and those of a control group in terms of their T2 star values.

**Study Design:**

Case-control study.

**Methods:**

Twenty patients diagnosed with pre-eclampsia and 22 healthy controls were included in this study. The placentas obtained after births performed via Caesarean section were taken into the magnetic resonance imaging area in plastic bags within the first postnatal hour, and imaging was performed via modified DIXON-Quant sequence. Average values were obtained by performing T2 star measurements from four localisations on the placentas.

**Results:**

T2 star values measured in the placentas of the control group were found to be significantly lower than those in the pre-eclampsia group (p<0.01). While the mean T2 star value in the pre-eclamptic group was found to be 37.48 ms (standard deviation ± 11.3), this value was 28.74 (standard deviation ± 8.08) in the control group. The cut-off value for the T2 star value, maximising the accuracy of diagnosis, was 28.59 ms (area under curve: 0.741; 95% confidence interval: 0.592-0.890); sensitivity and specificity were 70% and 63.6%, respectively.

**Conclusion:**

This study, the T2 star value, which is an indicator of iron amount, was found to be significantly lower in the control group than in the pre-eclampsia group. This may be related to the reduction in blood flow to the placenta due to endothelial dysfunction and vasoconstriction, which are important in pre-eclampsia pathophysiology.

Pre-eclampsia is one of the leading causes of maternal and perinatal mortality. Various factors, such as endothelial dysfunction, vasoconstriction, and oxidative stress, are described in the pathophysiology of pre-eclampsia. Although the aetiology has not been fully clarified, vascular dysfunction resulting in poor placentation is considered to be the main cause (1,2).

Iron, which is present in the composition of many body molecules, such as cytochrome, haemoglobin (Hb), and myoglobin, is stored as ferritin after it is absorbed by the mucosa of the small intestine. Fundamentally, iron is stored in the liver parenchyma and reticulo-endothelial system (3).

Magnetic resonance imaging (MRI) is the best non-invasive imaging method to reveal iron loading (4). Iron ions that accumulate in tissue can cause local distortion in the relaxation of spins and the body’s magnetic field due to their superparamagnetic features and thus cause T1 (longitudinal relaxation time), T2 (transverse relaxation time), and, especially, T2 star (T2*) (transverse relaxation time affected by magnetic field in homogeneity) shortening. This effect causes signal loss in the affected organ in proportion with the amount of iron stored (5,6). T2*-weighted images have the greatest sensitivity in terms of detecting iron (7). There are many reasons for iron loading in the body. The aim of this prospective study was to examine whether there is a difference between the placentas of pre-eclamptic pregnant women and those of a control group in terms of T2* values, which are a quantitative indicator of the amount of iron and are obtained via MRI.

## MATERIALS AND METHODS

### Design

Informed consents were obtained from all patients and approval for the study was granted by the Ethics Committee of Sütçü İmam University of Medical Sciences (No: 06, Date: 14 December 2015). The study included 20 pre-eclamptic placentas obtained from Caesarean section delivery and 22 healthy placentas as a control group. The mean maternal age was 28.3±5.131 years in the pre-eclamptic group and 31±5.033 years in the control group. Placentas with gross placental haematoma and gross calcification, placentas with multiple pregnancies, and placentas with foetal major chromosomal-structural abnormalities were excluded. Demographic features and detailed obstetric and medical data were collected from the hospital information system and prenatal care records. The 1st- and 5th-minute postnatal Apgar score, birth week, birth weight, and postpartum short-term medical status data were recorded. A diagnosis of pre-eclampsia was made according to the criteria of the American College of Obstetricians and Gynecologists (8). Pre-eclampsia was defined as hypertension (systolic to diastolic blood pressure ≥140/90 mm Hg) and proteinuria (>300 mg/day in 24-hour urine collection or a protein concentration of 1 g/L) that started during pregnancy and ended after the birth. The Hb and haematocrit (Hct) values from the complete blood cell count values measured within 24 hours of birth were recorded.

### Imaging technique

**FIG. 1.**
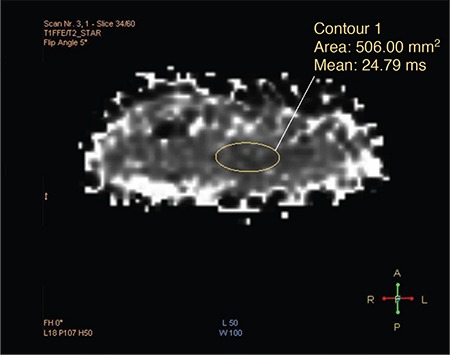
T2 star image of modified DIXON-Quant sequence demonstrates placement of ROI for measurement of the placenta.

Placentas were clamped at a standard height on the obstetric table after the birth, put in plastic bags, and taken to the MRI unit within one hour of birth. Screening was performed in a Philips Ingenia 1.5 T (Eindhoven, the Netherlands) machine using a SENSE Torso XL coil. Modified DIXON (mDIXON)-Quant sequences were obtained. The T1 FFE mDIXON-Quant sequence parameters were TR/TE1/delta TE 5.4/0.93/0.7 ms, six echoes, flip 5 deg, BH 6 sec, slice thickness 3 mm, matrix size 128x128, and field of view 250 mm. Images were evaluated on the workstation (IntelliSpace Portal Philips v6.03.13200 Philips Healthcare Nederland B.V, Veenpluis 4-6, 5684 PC Best, the Netherlands). The measurements were taken on T1FFE/T2* images, which are one of the four image types (T1FFE/W, T1FFE/F, T1FFE/FF, and T1FFE/T2*) obtained in a fast single sequence. The measurements were taken using a standard ROI (region of interest) (mean 500 mm2) from four fields to prevent over-sensitivity to artefacts where the maximum homogeneity was obtained (Figure 1), and the mean values were obtained.

### Statistical analysis

According to post hoc power analysis, using a mean T2* value parameter at alpha 0.05 level, test power achieved 83% with group sample sizes of 22 and 20. Data were expressed as mean±standard deviation (SD). The student’s t-test and Pearson correlation test were applied to detect correlations between groups. Receiver operating characteristic curves were applied to check the diagnostic value of T2* in the estimation of pre-eclamptic placentas and the best T2* cutoff value, which represents the diagnosis of pre-eclampsia. Sensitivity and specificity were calculated based on T2* measurements. Computer software (Statistical Package for the Social Sciences Version 15.0, SPSS Inc., Chicago, IL, USA) was used for statistical analysis. A value of p<0.05 was accepted as statistically significant.

## RESULTS

Maternal ages, birth weeks, birth weights, 1st- and 5th-minute Apgar scores, and maternal Hb and Hct values are shown in Table 1. There was no statistically significant difference between the groups in terms of mean maternal age, birth weight, 1st- and 5th-minute Apgar scores, or maternal Hb and Hct levels (p=0.093, p=0.105, p=0.543, p=0.492, p=0.128, and p=0.128, respectively). The mean urinary protein values of the pre-eclamptic group and the control group were 2.1±0.71 and 0.27±0.88, respectively. The mean systolic and diastolic tension values of the pre-eclamptic group and the control group were 162±10.05/98.5±7.45 and 121.82±20.61/67.73±7.45, respectively. As expected, the urinary protein and systolic and diastolic tension mean values were statistically significantly higher in the pre-eclamptic group (p<0.001 and p<0.001, respectively). There was no statistically significant correlation among the urinary protein, systolic and diastolic tension values, and placenta T2* values of the pre-eclamptic group (p=0.233, p=0.730, and p=0.148, respectively).

**FIG. 2.**
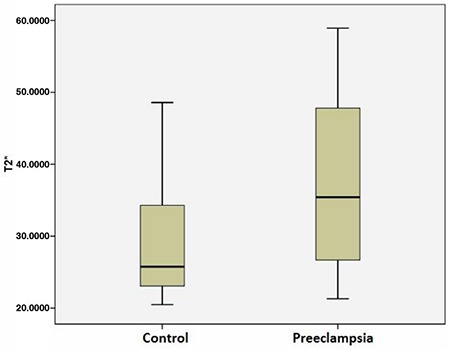
Box plot showing T2 star values of pre-eclamptic and control group placentas.

A statistically significant difference was detected between the two groups in terms of T2* values (p<0.01) (Figure 2). While the mean T2* value in the pre-eclamptic group was found to be 37.48 ms (SD±11.3), this value was 28.74 (SD±8.08) in the control group. The mean birth week of the pre-eclamptic and control groups was 35w 1d±2w 4d and 37w 6d±1w 2d, respectively. The birth week for the control group was significantly higher than that of the pre-eclamptic group (r=0.659, p<0.001). There was a strong negative correlation between the T2* values of all the placentas and the birth week (r=-0.510, p<0.001). There was a strong negative correlation between the birth week and the T2* values in the control group (r=-0.460, p<0.05), and no correlation between birth week and T2* values in the pre-eclamptic group (r=-0.337, p=0.147). The cutoff value for the T2* value, maximising the accuracy of diagnosis, was 28.59 ms (area under curve: 0.741; 95% confidence interval: 0.592-0.890); sensitivity and specificity were 70% and 63.6%, respectively.

## DISCUSSION

As a result of this study, which was conducted ex vivo and was the first MRI study measuring the amount of iron in the placenta, T2* values, which are the quantitative indicator of iron loading, were found to be higher in the pre-eclamptic group placentas than in the control group. Pre-eclampsia is a hypertensive dysfunction related to pregnancy-induced proteinuria, and it appears after the 20th gestational week. This dysfunction affects 7-10% of all pregnancies and is highly related to perinatal mortality and morbidity (9,10).

Although the pathophysiology of pre-eclampsia has not been clarified, genetic factors, abnormal placentation, endothelial dysfunction, and immunological interactions are described as possible pathophysiological mechanisms (11). It is known that vasoconstriction that occurs due to the increasing response to vasoactive substances, such as angiotensin II and endothelin, takes place in the pathophysiology of pre-eclampsia (12). Pre-eclampsia is characterised by vascular dysfunction, reduced systemic vasodilators, and increased uteroplacental resistance as a result of impaired extravillous trophoblast migration toward the uterine spiral arteries. In terms of its pathogenesis, tissue damage caused by an increase in the levels of reactive oxygen species and the altered bioavailability of nitric oxide (NO) plays an important role. Peroxynitrite, which is formed after superoxide quickly inactivates NO, accumulates in placental tissue and disrupts placental function. Moreover, reactive oxygen species may stimulate platelet adhesion and aggregation, causing intravascular coagulopathy. Ultimately, placental infarct increases and oxygen and nutrients decrease due to the impairment of the uteroplacental bloodstream, causing foetal growth retardation. On the other hand, antioxidants, such as catalase, superoxide dismutase, and glutathione peroxidase, clean reactive oxygen species and thus protect tissue against oxidative stress. Placental ischaemia causes oxidative stress to increase by reducing antioxidant activity, disrupting the balance between reactive oxygen types and antioxidant activity (13).

Iron, which is present in various body molecules, especially cytochrome, Hb, and myoglobin, is stored as ferritin after it is absorbed by the mucosa of the small intestine (3). Iron loading may develop due to the malabsorption of iron, such as via hereditary hemochromatosis, a dysfunction in heme metabolism, or long-term transfusion therapy (14). While surplus iron accumulates primarily in the liver, pancreas, heart, thyroid, pituitary, and synovium, in its secondary form, it is stored in the reticuloendothelial system (kupffer cells, spleen, bone marrow, and lymph nodes). When this system is saturated, iron is stored in hepatocytes and in other parenchymal cells (15). Surplus iron may catalyse the transformation of hydrogen peroxide into free radicals, and it may cause damage to cell membranes, protein, and DNA. Iron is toxic to tissues, and it may cause hepatic, cardiac, or endocrine function disorders. In order to monitor the response to treatment, quantifying iron content is important (3). Traditionally, this measurement was taken indirectly via serum ferritin level or directly via biopsy. A weak correlation has been established between iron stored in the organs and ferritin measurements via invasive biopsy (16).

MRI is a non-invasive method that can demonstrate surplus iron loading in various organs, such as the liver, heart, spleen, and pancreas, and quantify it, and the correlations obtained in this manner are typically sound. Recently, many quantitative MRI techniques have been presented. Most are T2*-weighted, gradient echo sequence-based, and obtained using progressively longer echo times (15). There are two methods used to quantify iron loading in the liver: signal intensity ratio methods and relaxometry methods. Whereas signal intensity ratio methods measure the signal intensity and noise of the tissue (17,18), relaxometry methods measure the signal intensity of the tissue for multiple echo times and thus calculate T2 or T2* values. T2 or T2* values are inversely proportional to iron concentration (19). T2* values under 18 ms are accepted as indicating overloading in the liver (20).

Dual-echo (2E-mDIXON), adapted from a modified DIXON (mDIXON) technique and allowing flexible echo times, is used as a fast and high-resolution fat suppression technique (21,22,23). Six-echo mDIXON (6E-mDIXON) is perfectly correlated with forecasted hepatocyte budget and enables an estimation of T2*decays (22,24,25).

As a result of normal, variant, or abnormal perfusion changes in the liver, iron loading in various forms, as well as various forms of protection from loading, have been described (15,26). In one study, in the fields in which segmental signal reduction was observed in the liver via MRI, angiography, and CT portography, the decay of portal flow and the presence of abnormal iron loading were demonstrated by confirming the pathological result (26). The current study aimed to show a difference in iron amount via MRI, which would suggest that there were differing iron amounts in the placentas of the pre-eclampsia group, where there was vasoconstriction and endothelial dysfunction due to perfusion changes. With the aim of performing a quantitative evaluation, the measurement of T2* values in this study was applied using the 6E-mDIXON sequence.

The results of this study showed that in pre-eclamptic placentas with perfusion dysfunction in their pathophysiology, the amount of iron was found to be less than that of the control group. While iron amounts differed between the groups, no difference was detected with regard to maternal Hb and Hct values. There was a difference between the birth weeks of the two groups, and in the in-group correlation analysis, there was a negative correlation between the T2* value and birth week of the control group (i.e., as the weeks progressed, the amount of iron in the placenta increased). No significant difference was determined in T2* values as birth weeks progressed in the pre-eclamptic group. This may be because of a reduced blood flow to the placenta due to endothelial dysfunction and vasoconstriction, which may have caused less iron loading in the placenta. Based on the results of this study, there is a need for further in vivo MRI studies in which the amount of iron in the placenta is measured. With the help of that sequence, which could be performed in a short time, and measurement of the iron amount, a non-invasive screening method could be used in the early diagnosis and follow-up of pre-eclampsia.

There have been various MRI studies of pre-eclamptic placentas (27,28). However, there are no studies that measure the amount of iron in the placenta via MRI. Thus, the values obtained in this study could not be compared with any in the literature.

This study has certain limitations. The control group was formed randomly. As expected, a difference was detected in the birth week for the pre-eclampsia and control groups. There was no histopathological evaluation of the placentas. Concurrent histopathological studies with MRI may reveal the relationship between structural placenta changes and the MRI findings. It is hoped that this article will encourage future in vivo MRI studies with larger patient groups and studies supported by histopathological correlation.

Iron loading is relatively common and has various causes. Information based on screening findings may provide useful data during the process of diagnosis and treatment. MRI is the best non-invasive screening method for quantitative measurement of the amount of iron. At the end of the current study, the iron amount in pre-eclamptic placentas was found to be lower than that of the control group. This could have developed secondarily to vasoconstriction and endothelial dysfunction. Further studies of larger patient groups and with histopathological confirmation are needed.

## Figures and Tables

**Table 1:**
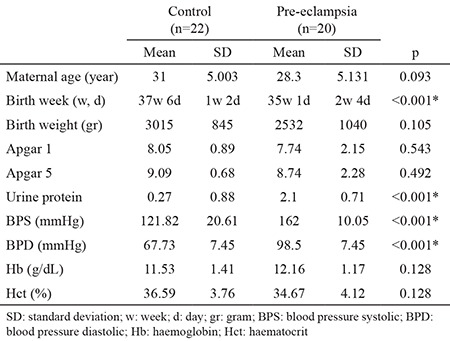
Obstetric and medical features of pre-eclamptic and control groups
